# Northern Nordic river mouth N:P:Si stoichiometry shows limited evidence of Si depletion

**DOI:** 10.1038/s41598-025-34052-w

**Published:** 2026-01-07

**Authors:** Martyn Futter, Katri Rankinen, Joachim Audet, Heleen de Wit, Csilla Farkas, Martin Forsius, Jens Fölster, Anastasija Isidorova, Øyvind Kaste, Dominika Krzeminska, Katarina Kyllmar, Emma Lannergård, James Sample, Eva Skarbøvik, Lars Sonesten, Brian Kronvang

**Affiliations:** 1 Department of Aquatic Sciences and Assessment, Swedish University of Agricultural Sciences, Uppsala, Sweden; 2 Finnish Environment Institute, Helsinki, Finland; 3 Aarhus University, Aarhus, Denmark; 4 Norwegian Institute for Water Research, Oslo, Norway; 5 Department of Biosciences, University of Oslo, Oslo, Norway; 6 Norwegian Institute of Bioeconomy Research, Ås, Norway; 7 Department of Soil and Environment, Swedish University of Agricultural Sciences, Uppsala, Sweden

**Keywords:** Biogeochemistry, Environmental sciences, Hydrology

## Abstract

River mouth nitrogen: phosphorus: silicon (N:P:Si) stoichiometry can predict marine eutrophication potential. Furthermore, deviations from Redfield molar N: P:Si = 16:1:20 ratios can offer insight into limiting nutrients and risks for harmful algal blooms (HAB). Here, we present N:P:Si stoichiometry based on total P, dissolved inorganic N and dissolved Si for 88 coastal river mouths in Norway, Sweden and Finland between 2017 and 2024. Rivers ranged from ultra-oligotrophic to hyper-eutrophic. N and P concentrations increased from north to south, with no latitudinal Si gradient. Most rivers were either P or jointly NP depleted relative to Si, with no overall evidence of Si depletion. However, there was some evidence of seasonal Si depletion. We show how using multi-element approaches, e.g., the Index of Coastal Eutrophication Potential (ICEP) can help to guide management actions. Specifically, Using Si depletion relative to N and P concentrations as an indicator of HAB potential may offer a means to identify catchments where nutrient load reductions can have the largest benefit on marine ecosystem health. Such multi-element approaches can complement single nutrient assessments based on, e.g., the Carlson Trophic Status Index or Water Framework Directive status class assessments.

**Supplementary Information:**

The online version contains supplementary material available at 10.1038/s41598-025-34052-w.

## Introduction

Element availability can be considered either in isolation or jointly with other elements. The latter is often known as stoichiometry. Ideally, stoichiometric analyses should consider both the relative proportions of different elements as well as their absolute concentrations. Most water quality management programmes set targets for individual nutrient concentrations (e.g., the EU Water Framework Directive, EC 2000/69) or loads (US Total Maximum Daily Loads^1^; Chinese water quality standards^2^. However, these single element approaches neglect environmentally relevant interactions including, e.g., limitation associated with other water quality parameters^3,4^. New approaches to managing water quality based on consideration of multiple elements simultaneously are needed to promote biological carbon storage^5^, limit greenhouse gas emissions^6^ and reduce the negative impacts of human activities on surface water eutrophication^3,4^.

Human activities perturb natural element cycles, which in turn influence element availability. Agricultural practices, particularly the widespread use of nitrogen (N) and phosphorus (P) fertilisers to increase food production, have markedly increased the fluxes of those elements from land to sea^7^. At the same time, changes in terrestrial vegetation as well as river regulation (damming) have likely decreased fluxes of silicon (Si) from land to sea^8–10^. This stoichiometric imbalance in the supply of N, P and Si can promote harmful algal blooms (HABs)^11^. When sufficient Si is available relative to N and P, siliceous taxa (i.e., diatoms and chrysophytes) can dominate the phytoplankton community. However, when there is a shortage of Si relative to other nutrients, other taxonomic groups, including toxic and harmful cyanobacteria can proliferate in marine and coastal environments^12^.

Eutrophication control is one area where a multi-element perspective may be useful. For example, either N or P can limit primary production^4^ and C can also be limiting, especially in eutrophic systems^13^. In addition to the macronutrients (C, N and P), micronutrients including iron, manganese and cobalt can potentially limit primary production^14^. Furthermore, Si can be a driver of phytoplankton community composition^15,16^.

There are long-standing debates about whether controlling N or P is more likely to lead to reductions in eutrophication^3,4^. The decision about which nutrient to control can be based on NP imbalances^4^. One approach to estimating and communicating NP imbalances and their potential for nutrient management has been to use the Redfield ratio^17^. This ratio is based on observations made in the 1930 s by Alfred Redfield of the stoichiometric composition of marine phytoplankton^18^. While most subsequent studies have focussed on C: N:P ratios^19–21^, Redfield also developed ratios for other elements including Si^22^.

The Redfield C:N:P molar ratio of 106:16:1 is well known and widely used in aquatic science and elsewhere as an indicator of nutrient depletion and potential nutrient limitation^19–21^. Ternary diagrams are widely used in soil sciences to represent the proportions of three substances (e.g., sand, silt and clay) when the proportions sum to one. Ternary diagrams have been used to show deviations from Redfield ratios^19,20^ based on proportions of three elements relative to the Redfield ratio. Redfield diagrams have typically been used for showing C:N:P stoichiometry but could in principle be used to represent any three-component system. To the best of our knowledge, they have not yet been used for presenting N:P:Si stoichiometry.

Multiple values have been proposed and used for N:P:Si molar stoichiometric ratios, e.g.,16:1:16^12,22^, 16:1:17^23^, 16:1:20^15,16^ and 16:1:40^24^. While the N: P molar ratio is 16:1 in all cases, there is significant variation in the Si values used. The different values represent Si demand by siliceous producers where higher Redfield Si values are appropriate for freshwaters and lower values are appropriate for marine systems. Different forms of N, P and Si have been used for N:P:Si Redfield stoichiometry assessments. Most assessments have been based on measurements of total P while measurement of both total^15^ and dissolved inorganic N (DIN)^11,16^ have been used. While both dissolved and particulate Si are present in terrestrial surface waters, Billen and Garnier^15^ proposed that the dissolved fraction, i.e., DSi, should be used as it is the fraction that is readily bioavailable.

Following Moore et al.^25^, we define nutrient depletion as “the stoichiometric lack of one element relative to another”. Nutrient limitation occurs when the one or more nutrients are present in levels such that either the growth of individual cells or production of new biomass is constrained^25^. Nutrient depletion is an indicator of potential limits to phytoplankton growth as opposed to empirical evidence of growth limitation. While nutrient limitation implies nutrient depletion, the reverse is not necessarily true (i.e., nutrient depletion may not lead to nutrient limitation as growth and maintenance potential may not be affected).

Eutrophication control measures are more likely to be effective when they address the limiting nutrient Identifying whether N or P is likely to be limiting primary productivity can help to target nutrient reduction measures^4^. Billen and Garnier^15^ were amongst the first to identify the importance of Si in eutrophication assessments. They developed an index of coastal eutrophication potential (ICEP) relating river mouth N, P and Si fluxes to marine eutrophication. The ICEP is based on the observation that HABs are less frequent when sufficient Si is available to support growth of siliceous phytoplankton. They hypothesized that when waters become Si depleted but there is still available N or P, blooms of undesirable taxa can occur^15^. Thus, ensuring that N and P inputs are balanced relative to Si may reduce the incidence of adverse water quality events associated with eutrophication. This index has received widespread use and contributes to assessments of progress towards achieving the UN Sustainable Development Goal (SDG) 14.1.1a^26^ related to coastal eutrophication.

Here, we present an analysis of average multi-year (2017–2024) N:P:Si stoichiometry in northern Nordic river mouths. We explore the use of critical N and P threshold concentrations based on Si levels for making informed decisions about catchment nutrient management and discuss choice of nutrient fraction for stoichiometry analysis. We conclude with recommendations for the use of nutrient stoichiometry in management decision making.

## Methods

### Data sources

**Fig. 1:**
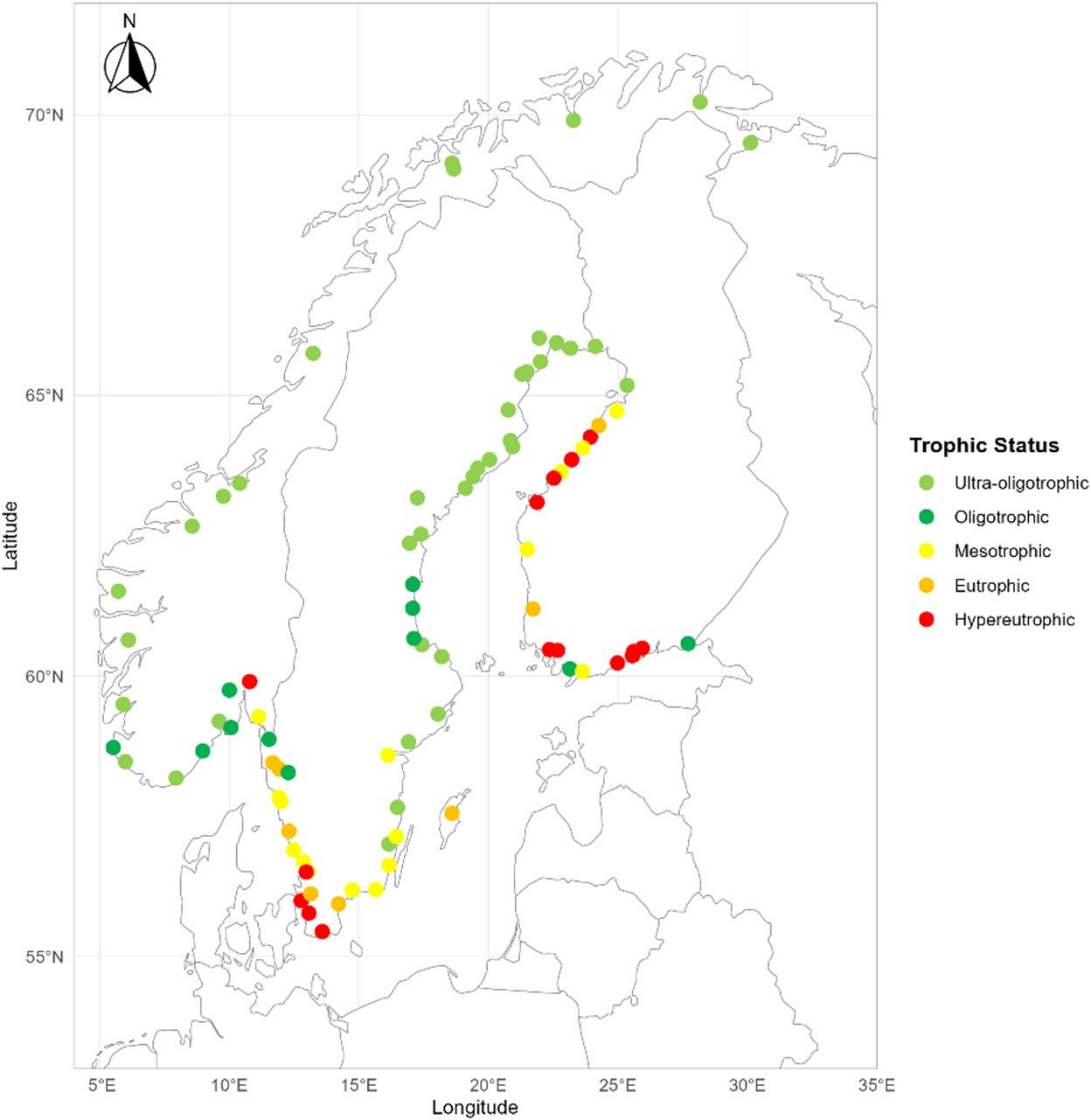
Locations of river mouth monitoring sites in Norway, Sweden and Finland. Locations are colour coded by the trophic status index (TSI) class of the Redfield depleted nutrient (i.e., molar N:P < 16 uses TSI(DIN), molar N:P > 16 uses TSI(TotP) Light green represents ultra-oligotrophic conditions (TSI < 30), dark green shows oligotrophic river mouths (30 < TSI < 40), yellow represents mesotrophic conditions (40 < TSI < 50) while orange indicates eutrophic conditions (50 < TSI < 60) and red indicates hypereutrophic conditions (TSI > 60).

Water chemistry data were obtained from Finnish, Swedish and Norwegian long-term river mouth monitoring programmes (Fig. 1, Supplementary Table 1). These programmes collect monthly samples for water quality analysis conducted to support two European Regional Seas programmes: OSPAR, the Convention for the Protection of the Marine Environment of the North-East Atlantic and HELCOM, the Baltic Marine Environment Protection Commission. As Denmark does not routinely measure Si in river mouth monitoring programmes, it was not included. In the following analyses, we used a Redfield N:P:Si molar ratio of 16:1:20 for consistency with SDG reporting^26^.

The Swedish river monitoring programmes have been operational since the mid 1960s^27^. Data from 55 locations was available for analysis. Rivers were included in either the river mouth monitoring programme^28^ or the Trend Watercourses monitoring programme^29^. Surface (< 1 m) samples were used in all cases. Total P and total Si measurements were used to represent P and Si respectively. Dissolved inorganic nitrogen (DIN) was calculated as the sum of nitrate + nitrite and ammonia + ammonium. Most northern Swedish rivers have hydropower reservoirs in their catchments and others, e.g., Stockholm Centralbron, Nyköpingsån and Göta älv, all drain large lakes.

Data from the Norwegian monitoring programme for nutrient inputs to coastal regions were available for some rivers starting in 1990. The monitoring programme was expanded at the start of 2017 to include rivers draining minimally impacted catchments, giving a total of 21 rivers. In the monitoring, total P, nitrate, ammonia and SiO_2_ are measured at monthly intervals. For the analyses presented here, DIN was calculated as the sum of nitrate plus ammonia. The Norwegian monitoring reported both Si and SiO_2_. When SiO_2_ was reported and Si was not available, Si was estimated from SiO_2_ by multiplying the reported SiO_2_ by the molar mass of Si (28.08 g mol^− 1^) divided by the molar mass of SiO_2_ (60.08 g mol^− 1^). Additional information about the Norwegian monitoring programme is available^30^.

Data from twenty Finnish rivers were available. In most cases, data were available from the 1990 s to the present. The selection of rivers presented here are from the national monitoring programme of Finnish waters^31,32^. Unlike the Swedish rivers, the Finnish rivers analysed here had no large lakes or lake chains in their catchment and no significant point sources of nutrients. Measurements of total P, DIN (the sum of nitrate and ammonia) and dissolved SiO_2_ were used in the analysis presented here. River mouth sites were retained for further analysis if they had a minimum of six observations per year for the period of 2017 to 2024.

More information about the Swedish and Finnish catchments is available in the HELCOM metadata catalogue^33^.

Additional regional data were obtained from published sources and open access databases. For the North Sea, UK data for 39 rivers were obtained from published sources^12^. Baltic Sea Region river mouth data were obtained from the Gemstat data portal^34^ and the Danish national monitoring programme NOVANA^35^. Data for the Nemunas River in Lithuania were obtained from^36^. While the Gemstat database includes coastal rivers in Poland, Estonia, Germany and Lithuania, no Si measurements were available for these locations.

### Ternary diagrams

Ternary diagrams have a long history in environmental science, primarily for presenting sand/silt/clay proportions in soil. However, they have also been used for presenting element stoichiometry in other systems including plants (N:P:K)^37^ and rivers (C:N:P)^19^.

A ternary diagram presents the proportions of three factors. Factor values range between 0 and 1, and sum to 1. When using ternary diagrams to show deviations from the Redfield ratio, it is necessary to scale all observations so that a water chemistry sample with an N: P:Si molar ratio of 16:1:20 will plot at the centre of the ternary diagram at coordinates 33.3%, 33.3%, 33.3%^20^. This can be accomplished using Eqs. 1–3 where DIN, TotP and Si are the molar concentrations of N, P and Si respectively with units of µmol l^− 1^. In all ternary plots presented here, zones of nutrient depletion are arbitrarily identified as locations where Redfield % DIN, TotP or Si are less than 20% (Supplementary Fig. 1).

1 $${\text{Redfield\_N}}\% {\text{ }}={\text{ }}100{\text{ }}\operatorname{x} {\text{ }}\left( {{\text{DIN}}/16} \right){\text{ }}/{\text{ }}({\text{DIN}}/16\,+\,{\text{TotP}}\,+\,{\text{Si}}/20)$$

2 $${\text{Redfield\_P}}\% {\text{ }}={\text{ }}100{\text{ }}\operatorname{x} {\text{ TotP }}/{\text{ }}({\text{DIN}}/16\,+\,{\text{TotP}}\,+\,{\text{Si}}/20)$$

3 $${\text{Redfield\_Si}}\% {\text{ }}={\text{ }}100{\text{ }}\operatorname{x} {\text{ }}\left( {{\text{Si}}/20} \right){\text{ }}/{\text{ }}({\text{DIN}}/16\,+\,{\text{TotP}}\,+\,{\text{Si}}/20)$$

### Calculations

The Carlson trophic status index (TSI)^38^ is widely used as an indicator of waterbody eutrophication status. TSI values can be estimated for nutrients (N and P), chlorophyll concentrations and transparency (Secchi depth). Carlson^39^ recommends the use of a single TSI to describe waterbody status as opposed to composite or summary indices. Here, we used TSI values of the more depleted nutrient (N or P) as an indicator of eutrophication status. TSI values for P (Eq. 4) were calculated using^39^. For N, TSI (Eq. 5) was calculated using^40^. Both TotP and DIN have units of µmol l^− 1^. TSI(TotP) was used as an index of river mouth trophic status when there was evidence of P depletion (i.e., molar N: *P* > 16), otherwise, TSI(DIN) was used.

4 $${\text{TSI}}\left( {{\text{TotP}}} \right)\,=\,4.15\,+\,14.42{\text{ }}\operatorname{x} {\text{ ln}}\left( {31{\text{ }}\operatorname{X} {\text{ TotP}}} \right)\qquad{\text{N:P}}\,>\,16$$

5 $${\text{TSI}}\left( {{\text{DIN}}} \right)\,=\,54.45\,+\,14.43{\text{ }}\operatorname{x} {\text{ ln}}\left( {14{\text{ }}\operatorname{X} {\text{ DIN}}/1000} \right){\text{ N:P}}\,<\,16$$

TSI scores facilitate comparison of the eutrophication potential of N and P. Lower scores are indicative of more oligotrophic (nutrient poor) conditions while higher numbers indicate more eutrophic conditions.

The Index of Coastal Eutrophication Potential (ICEP) is an indicator of potential rate of undesirable phytoplankton growth expressed in units of kg C km^− 2^ d^− 1^ based on fluxes of N, P and Si through a river mouth^15,16^. The ICEP values for N and P are calculated as follows (Eqs. 6 and 7):

6 $${\text{ICEP}}\left( {\text{P}} \right){\text{ }}={\text{ }}[{\text{PFlx}}/31-{\text{SiFlx}}/\left( {28{\text{ }}\operatorname{x} {\text{ }}20} \right)]{\text{ x }}106{\text{ x }}12\qquad{\text{ N:P}}\,>\,16$$

7 $${\text{ICEP}}\left( {\text{N}} \right){\text{ }}={\text{ }}[{\text{NFlx}}/\left( {14{\text{ x }}16} \right)-{\text{SiFlx}}/\left( {28{\text{ x }}20} \right)]{\text{ x }}106{\text{ x }}12\qquad{\text{ N:P}}\,<\,16$$

NFlx, PFlx and SiFlx are the daily fluxes of N, P and Si per km^2^ catchment area and N: P is the molar N: P ratio. Positive ICEP values are indicative of potential for HAB production while negative values are indicative of conditions with sufficient Si relative to nutrients to potentially suppress HAB production. ICEP values are calculated based on the Redfield depleted nutrient, i.e., ICEP(N) is used when molar N: P ratios are less than 16, otherwise ICEP(P) is used.

An ICEP value of 0 identifies the threshold at which nutrient and Si inputs are balanced and no HAB production is expected^41^. This insight can be used to determine critical N and P concentrations below which HABS are not expected based on N: P:Si stoichiometry. As flux is equal to concentration times runoff, Eqs. 6 and 7 can be rearranged to estimate critical N and P concentrations (DIN_0 and TotP_0) which would give ICEP values of 0.

8 $${\text{TotP\_0}}\,=\,\left( {31{\text{ }}/{\text{ }}\left( {28{\text{ x }}20} \right)} \right){\text{ x Si}}$$

9 $${\text{DIN\_0 }}={\text{ }}\left( {\left( {14{\text{ x }}16} \right)/\left( {28{\text{ x }}20} \right)} \right){\text{ x Si}}$$

In Eqs. 8 and 9, DIN_0, TotP_0 and Si are all expressed in the same concentration units (µg l^− 1^). In practice, this means DIN_0 is equal to 0.4 times the Si concentration and TotP_0 is equal to approximately 0.055 times the Si concentration.

Deviations from critical concentrations (Eqs. 10 and 11) were standardized by dividing the measured concentration for the respective nutrient by the critical concentrations derived from N: P:Si stoichiometry (Eqs. 8 and 9).

10 $${\text{D\_DIN}}\,{\text{=}}\,{\text{DIN }}\left( {{\text{measured}}} \right){\text{ }}/{\text{DIN\_0}}$$

11 $${\text{D\_TotP}}\,{\text{=}}\,{\text{TotP }}\left( {{\text{measured}}} \right){\text{ }}/{\text{TotP\_0}}$$

Redfield ratios, critical N and P concentrations and deviations from critical concentrations were calculated for each sample individually. Values were then aggregated to annual or monthly averages, which were then aggregated to overall averages.

All calculations and data management were performed in Microsoft Excel and Microsoft Access.

## Results

### Average river mouth nutrient concentrations

Sufficient data were available to assess N:P:Si stoichiometry in 88 river mouths: 20 in Finland, 21 in Norway and 47 in Sweden (Fig. 1, Supplementary Table 1). Average concentrations and their standard deviations were 583 ± 982 µg l^− 1^ for DIN, 32 ± 33 µg l^− 1^ for TotP and 2702 ± 1485 µg l^− 1^ for Si for river mouth measurements made between 2017 and 2024. Nutrient concentrations generally followed a North: South gradient with higher concentrations in the South than in the North. (Fig. 1, Supplementary Table 1). Trophic status indices based on the more depleted element (i.e., TSI(DIN) for molar N: < 16 and TSI(TotP) for molar N:P > 16) ranged from ultra-oligotrophic to hyper-eutrophic. In general, rivers draining into the Eastern side of the Baltic (Finland) had higher nutrient levels than rivers draining from the west (Sweden). There was no latitudinal gradient in Si concentrations (not shown). Overall, DIN comprised 45% +/20% of the total N.

### Overall stoichiometry patterns

**Fig. 2:**
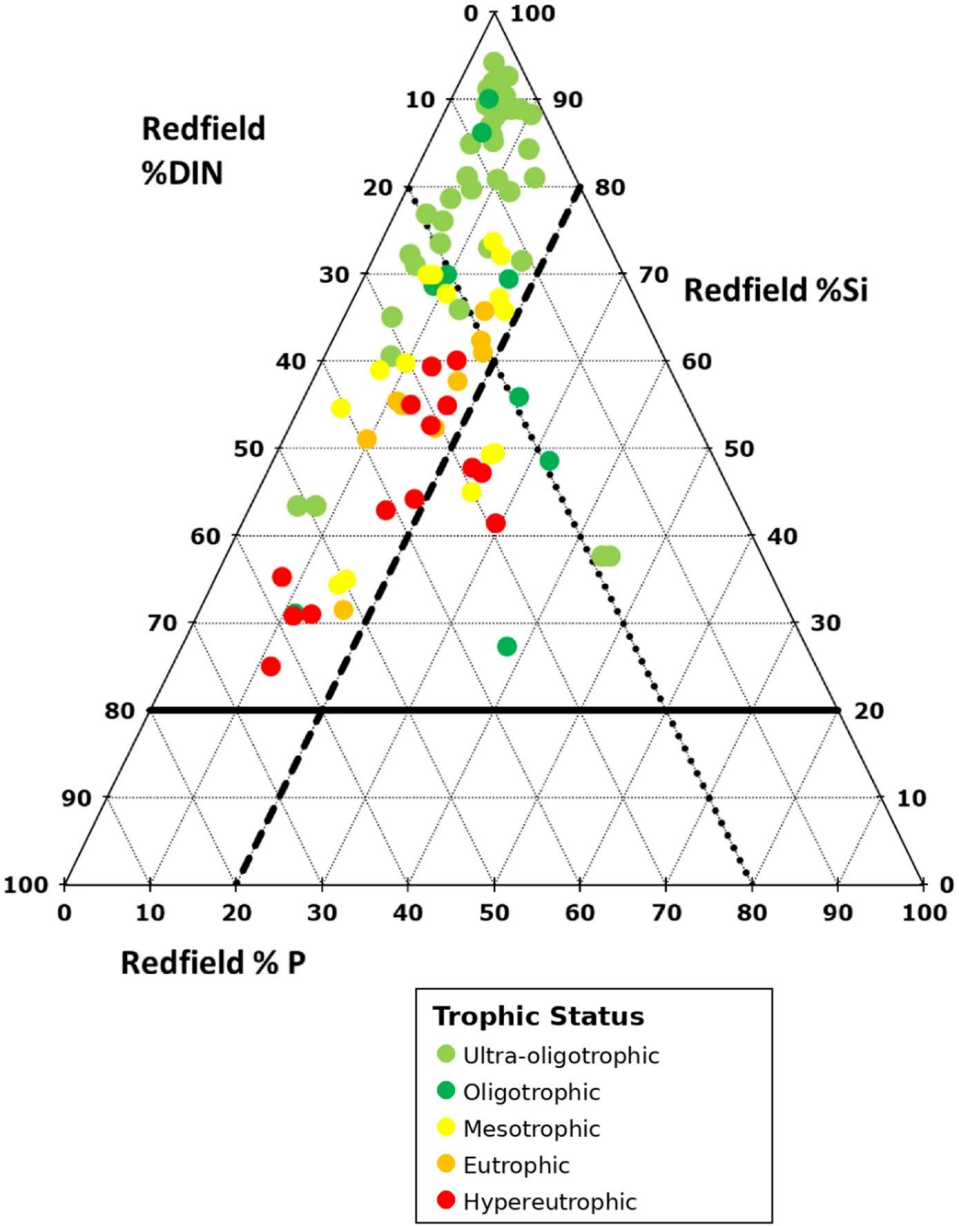
Average (2017–2024) molar N: P:Si ratios relative to the16:1:20 Redfield ratio observed at Nordic river mouths. Locations are colour coded according to the trophic status index (TSI) of the Redfield depleted nutrient (i.e., molar N: *P* < 16 reports TSI(DIN), molar N: *P* > 16 reports TSI(TotP)). Light green represents ultra-oligotrophic conditions (TSI < 30), dark green shows oligotrophic river mouths (30 < TSI < 40), yellow represents mesotrophic conditions (40 < TSI < 50) while orange indicates eutrophic conditions (50 < TSI < 60) and red indicates hypereutrophic conditions (TSI > 60).

Overall arithmetic average river mouth N:P:Si values and their deviations from the Redfield ratio were calculated for all Nordic river mouth monitoring locations based on measurements made between 2017 and 2024 (Fig. 2). There was a relationship between Redfield ratios and trophic status, with more oligotrophic river mouths typically showing N, P or joint NP depletion and more eutrophic sites typically showed sufficient levels of N and/or P relative to Si.

Most rivers (*n* = 77) were P depleted relative to the N: P:Si Redfield ratio. Of these, 39 showed evidence of joint NP depletion (plotting in the diamond at the top of Fig. 2). Most of these rivers are in northern Sweden as well as Norwegian rivers draining to the Arctic Ocean. Three rivers in Sweden and one in Finland were N depleted relative to P and Si. Four rivers in Finland, two in Sweden and one in Norway fell into the “balanced” Redfield zone at the centre of the plot where each nutrient had a Redfield percentage > 20%. No rivers showed evidence of overall Si depletion (i.e., Redfield Si percentage < 20%).

**Fig. 3:**
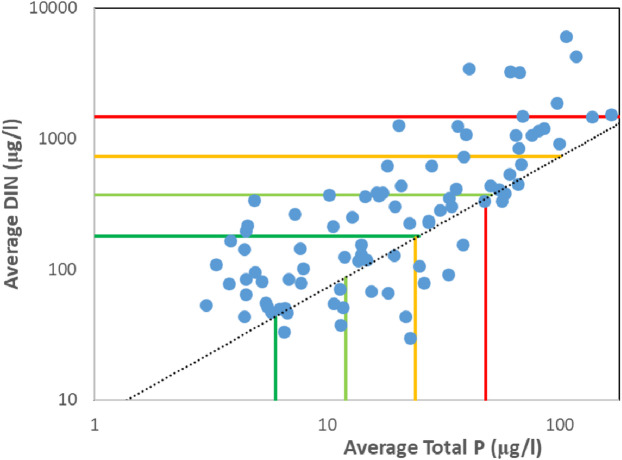
Average total P and DIN concentrations for the study rivers between 2017 and 2024. Blue dots represent individual river mouths. Vertical lines represent Carlson Trophic Status Index (TSI) boundaries while horizontal lines represent TSI boundaries for DIN. Values to the left or below of the dark green line are consistent with ultra-oligotrophic conditions. Values between the light green and dark green lines are consistent with oligotrophic conditions. Values between the yellow and light green lines are consistent with mesotrophic conditions. Values between the red and yellow lines are consistent with eutrophic conditions. Values to the right or above the red line are consistent with hypereutrophic conditions. The dotted black line represents the Redfield N: P ratio.

River mouths displayed a range of average DIN and total P concentrations (Fig. 3), spanning ultra-oligotrophic to hyper-eutrophic conditions. Thirty-four rivers were N enriched (DIN:TotP > 16) while the remaining 54 were N depleted (DIN:TotP < 16). There was no apparent relationship between nutrient concentrations and relative N or P enrichment. Note that the N:P ratio, and its implications for nutrient limitation is independent of Si.

**Fig. 4:**
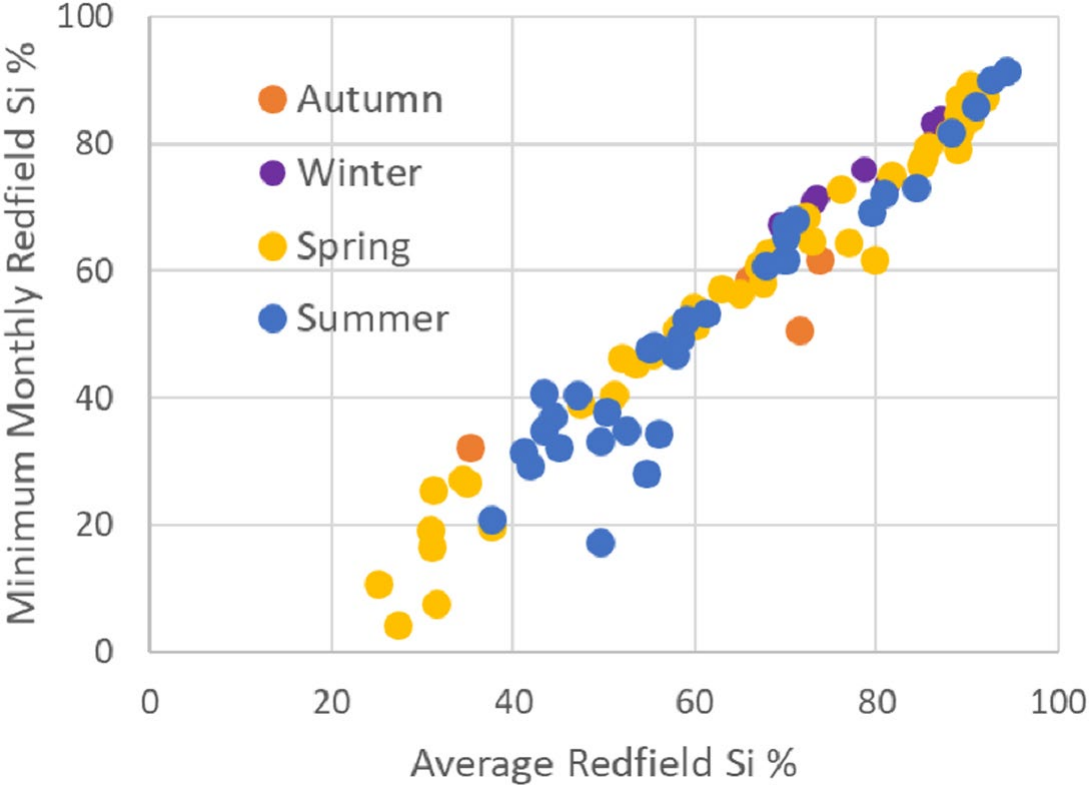
Overall average Redfield Si percentage versus minimum monthly Redfield Si percentage based on river mouth observations made between 2017 and 2024. Months in which minima occur are coded by season. Spring includes March-May (yellow), summer includes June-August (blue), autumn includes September-November (orange) and winter includes December-February (purple).

Redfield values for the month most depleted in Si suggested potential seasonal Si depletion in seven rivers (Fig. 4, minimum monthly Redfield Si < 20%). Four rivers had minimum Redfield Si percentages in autumn (September-November), 44 in spring (March-May), 33 in summer (June-August) and the remaining eight in winter.

### Exceedances of critical concentrations

**Fig. 5:**
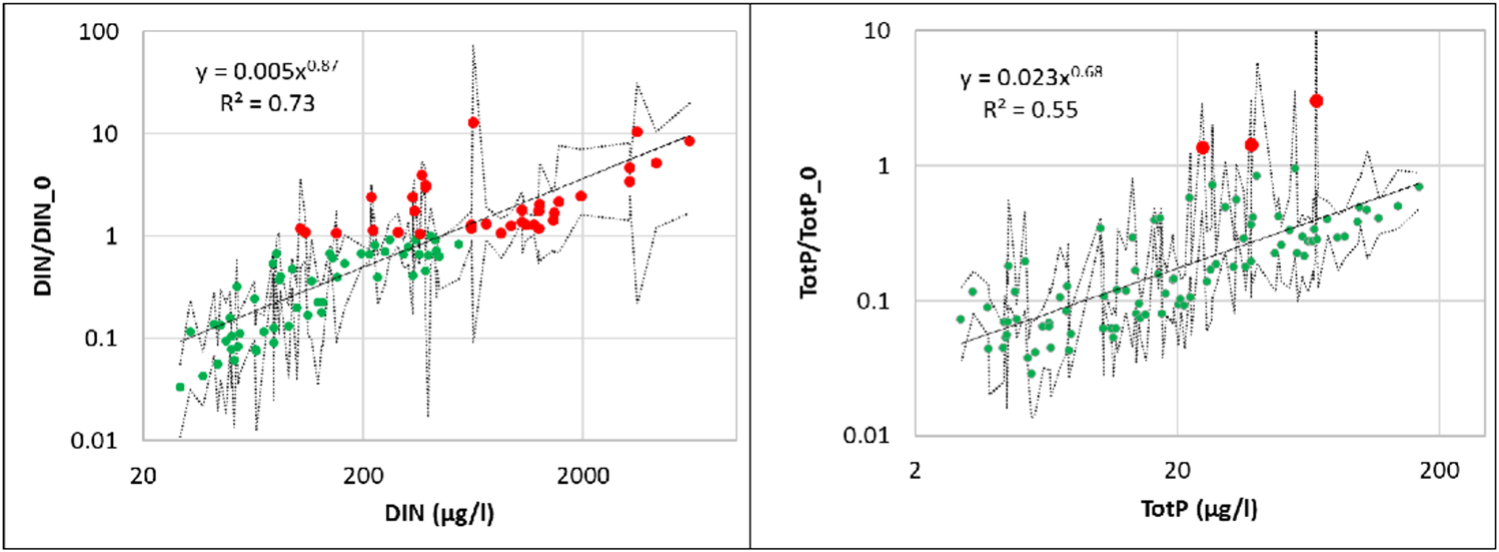
Exceedance ratios versus measured concentrations for DIN (left) and TotP (right). Dots show the overall average exceedance ratio for each river mouth. Dashed lines represent maximum and minimum monthly exceedance ratios. Green dots are indicative of rivers mouths with overall average exceedance ratios less than one while red dots indicate rivers mouths having average exceedance ratios greater than one.

Critical concentration values (Eqs. 8 and 9) for DIN and TotP were calculated for overall average and monthly values based on the relevant Si concentration measurements. Critical concentration exceedances (Eqs. 10 and 11) were estimated using the appropriate overall or monthly values. Exceedances were observed for both DIN and TotP (Fig. 5). While there was a monotonic relationship between exceedances and measured concentrations, there are some patterns worthy of further examination. DIN exceedances were not observed when average measured concentrations were less than approximately 100 µg l^− 1^. This value agrees well with the Reynolds nutrient limitation threshold^42^. For DIN concentrations between 100 and approximately 550 µg l^− 1^, average exceedance ratios above and below the critical threshold were observed. It was only when overall average DIN concentrations exceeded approximately 550 µg l^− 1^ that exceedance ratios greater than one (and hence positive ICEP value consistent with HAB production) were always observed. Almost all average overall measured TotP concentrations were less than the critical concentration. Only three river mouths, Orreelva in Norway, Nyköpingsån and Stockholm Centralbron, both in Sweden, had average TotP exceedance values greater than one.

### Broader regional patterns

In general, the N: P:Si stoichiometry values estimated for other rivers mouths elsewhere in the Nordic/Baltic region showed more evidence of Si depletion than northern Nordic river mouths (Supplmentary Fig. 2). This was especially true of UK rivers flowing into the North Sea and the Neva (Russia) and Nemunas rivers (Lithuania) which are major inflows to the Eastern Baltic. Nutrient stoichiometry in Latvian rivers was similar to stoichiometry reported here for Nordic rivers insofar as they had lower Redfield P percentages than N or Si.

## Discussion

Average N and P concentrations between 2017 and 2024 in northern Nordic river mouths span a range of Carlson TSI categories from ultra oligotrophic to hyper eutrophic. Regardless of N or P concentrations, river mouth chemistry was not Redfield Si depleted, i.e., average Redfield Si percentages are always greater than 20% and in most cases river mouth chemistry is N, P or jointly NP depleted relative to Si using an N: P:Si molar stoichiometry of 16:1:20. Based on the thresholds that are currently used in SDG reporting^15,16,26^, this would suggest that multi-year average Nordic river mouth N: P:Si stoichiometry has little or no potential for supporting HABs as river mouth chemistry is not Si depleted. This conclusion is based on average N: P:Si stoichiometry between 2017 and 2024 and average observed versus critical nutrient concentrations (i.e., the concentrations that are multiplied by flow to obtain ICEP values). That HABs are still occurring in the North and Baltic Seas^43^ suggests a more nuanced approach to element stoichiometry would be appropriate.

There are three possibilities to refine the use of river mouth element stoichiometry as an indicator of marine eutrophication. These are: using water chemistry measurements more related to bioavailable fractions, considering sub-annual (e.g., monthly) stoichiometry values and re-evaluating the terms in the Redfield ratio.

In the analysis presented here, we used total P and DIN. This is a compromise based both on data availability and the purpose of this study. Unlike Peacock et al.^19^ who were focussed primarily on catchment processes and their consequences for CNP fluxes to the marine environment, we were interested in evaluating whether or not river mouth nutrient stoichiometry could give insights into bioavailable nutrients and their possible controls on marine eutrophication. Total P likely overestimates the bioavailable fraction (i.e., some fraction of the P is recalcitrant) Much of the P in Nordic surface waters can be present in organic forms^44^. Using phosphate in Redfield ratio calculations would suggest that river mouths were even more P depleted while the use of total N would suggest lower levels of N depletion than presented here. Phosphate would also be problematic as it is present at levels close to the detection limit in many Swedish rivers^45^. DIN may underestimate bioavailable N as some but not all, of the organic N fraction is likely available for uptake by phytoplankton^46,47^. However, our use of DIN and total P is consistent with earlier work used in developing the ICEP indicator^16^.

Using different nutrient fractions would change the positions of individual river mouths on the ternary plot. If phosphate were used instead of total P, rivers would appear to be relatively more P depleted. Using total N instead of DIN would have the same effect, as N concentrations would be higher, rivers would appear to be more P depleted. Using phosphate instead of total P would make rivers appear less Si depleted whereas the use of total N versus DIN would have the opposite effect.

Future studies should explore sub-annual stoichiometric patterns. Monthly or seasonal estimates of nutrient depletion may be a better indicator of undesirable ecological conditions than overall or average conditions. The ICEP (i.e. the potential for eutrophication of coastal waters) provides an estimate of the mass of carbon that could be fixed by undesirable, non-diatomaceous, phytoplankton^16^. The seasonality of light and temperature regimes means that some months are more conducive to phytoplankton growth than others. For example, if Si depletion occurs in the summer, there may be a greater risk of marine HABs as northern seas are warmer and days are longer so conditions are less likely to be light or temperature limited.

The Redfield molar N:P:Si ratio of 16:1:20 as an indicator of marine eutrophication potential should be re-evaluated for Nordic conditions. The Baltic has a pronounced salinity gradient, ranging from near freshwater (practical salinity units (PSU) equal to zero to haline conditions with a PSU = 34 consistent with marine salinity at the outflow 2020^48^. The N:P:Si stoichiometry of diatoms can vary between 16:1:16 in marine environments^12,22^ to 16:1:40 in freshwaters^24^. While a Redfield molar ratio of 16:1:20 is relevant for reporting ICEP values as indicators of progress towards achieving the SDGs^26^, it may be less useful as a regional index of eutrophication. Using a Redfield ratio scaled to salinity (e.g., 16:1:40 for near freshwater conditions at the north of the Baltic and 16:1:16 at the outlet) might give a more appropriate indicator of marine eutrophication potential associated with terrestrial inputs from river mouths.

One potential drawback of the ICEP statistic is that it requires flux calculations instead of just water quality measurements. However, estimating critical concentrations based on N:Si and P:Si ratios instead of calculating ICEP values has both benefits and drawbacks. Concentration data can be used without any need to first calculate loads and that critical concentrations can identify possible targets for remediation. However, loads can be more useful when considering terrestrial impacts on the marine environment.

Regional patterns in stoichiometry are related to spatial patterns in N, P and Si concentrations and largely reflect differences in geology, soils, hydrological regimes, climate and land use. Riverine dissolved Si levels follow a north to south gradient around the Baltic Sea drainage basin, with higher concentrations in northern rivers^49^. In rivers draining northern alpine and sub-Arctic catchments the Redfield ratio is more NP depleted compared to in southern agricultural and urban dominated catchments where N and P concentrations are higher.

To control phytoplankton blooms, reductions in the more depleted nutrient are more likely to be successful^4^. The plots presented here may be useful to support decision making as they document regions where N, P and Si are either balanced or depleted. Further controls on the appropriate nutrient in river mouths showing evidence of Si depletion could be a more effective means of marine eutrophication control than strategies evaluating each nutrient in isolation, as is done with the EU Water Framework Directive and is implicit in the Carlson trophic state approach.

The present study did not attempt to address long-term trends in nutrient stoichiometry. In the 2017–2024 study period, there is little evidence of trends in Si concentrations, but over the longer term, increases in Si concentrations have occured in Norwegian lakes^50^. Furthermore, land use change could also influence riverine Si concentrations over time^10^. Changes in analytic methodology elsewhere in the study region may add complexity to any interpretation of long-term trends^51^.

Water chemistry is a useful indicator of eutrophication potential and quantifies pressures on the environment. It is not the same as measurements of actual impacts, e.g., HABs associated with river mouth inputs to the marine environment. Future work should attempt to “ground truth” these findings against observations of HABs offshore from suspect river mouths.

Incorporating Si into routine river mouth monitoring programmes throughout the OSPAR/HELCOM countries would give a more complete picture of eutrophication impacts. Currently, Si is routinely monitored in Norway, Sweden and Finland, as well as the UK^12^. River mouth Si measurements are not routinely reported by Denmark or Poland. Some data are available from Lithuania^36^, Latvia^52^ and Estonia^53^ but Si does not appear to be routinely monitored in river mouths. In general, rivers mouths draining into the Eastern and Southern Baltic are more Si depleted than northern Nordic river mouths. This is due to a combination of Si-poor bedrock geology and higher rates of N and P leaching associated with urban and agricultural areas. UK river mouths show a range of nutrient stoichiometries but are in general more N enriched than northern Nordic rivers.

Incorporating stoichiometric ratios into water quality management could potentially improve outcomes related to HABs and marine eutrophication. Focussing on nutrient reduction in Si-limited rivers should give more ecologically meaningful outcomes than current single element approaches. Classifying river mouth water nutrient chemistry as acceptable or unacceptable based on exceedance of critical concentrations demarcating the Redfield threshold for HABs could support more effective NP nutrient management in the Baltic and North Sea drainage basins.

## Supplementary Information

Below is the link to the electronic supplementary material.

Supplementary Material 1

## Data Availability

Data presented here are available at [https://doi.org/10.6084/m9.figshare.28389035.v1].
