# Supplementary material for: Northern Nordic river mouth N:P:Si stoichiometry shows limited evidence of Si depletion
Source: Sci Rep. 2026 Jan 7;16:884. doi: 10.1038/s41598-025-34052-w (PMC12783807; doi:10.1038/s41598-025-34052-w)
Supplement: Supplementary file 1 — Supplementary Material 1 [file 41598_2025_34052_MOESM1_ESM.docx]

| 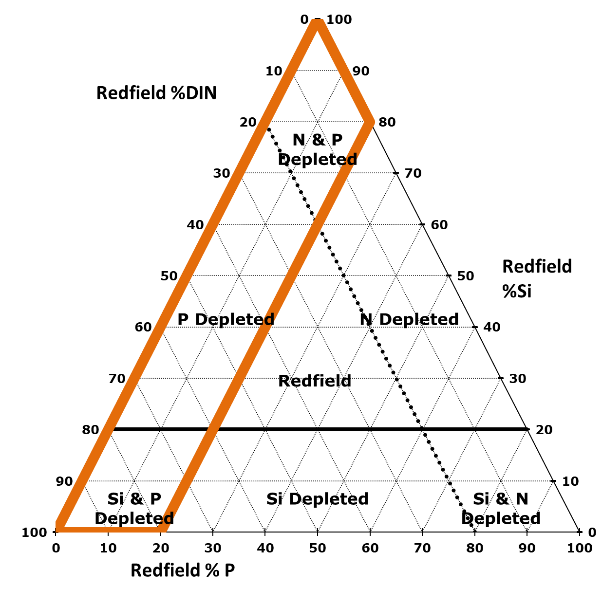 | 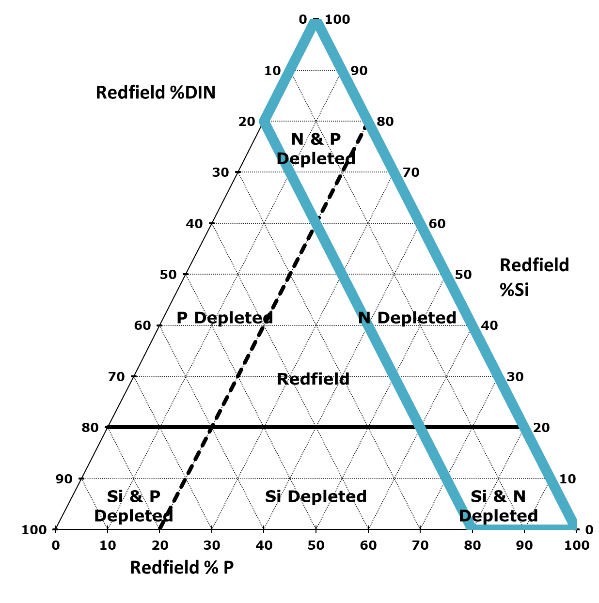 |
| --- | --- |
| 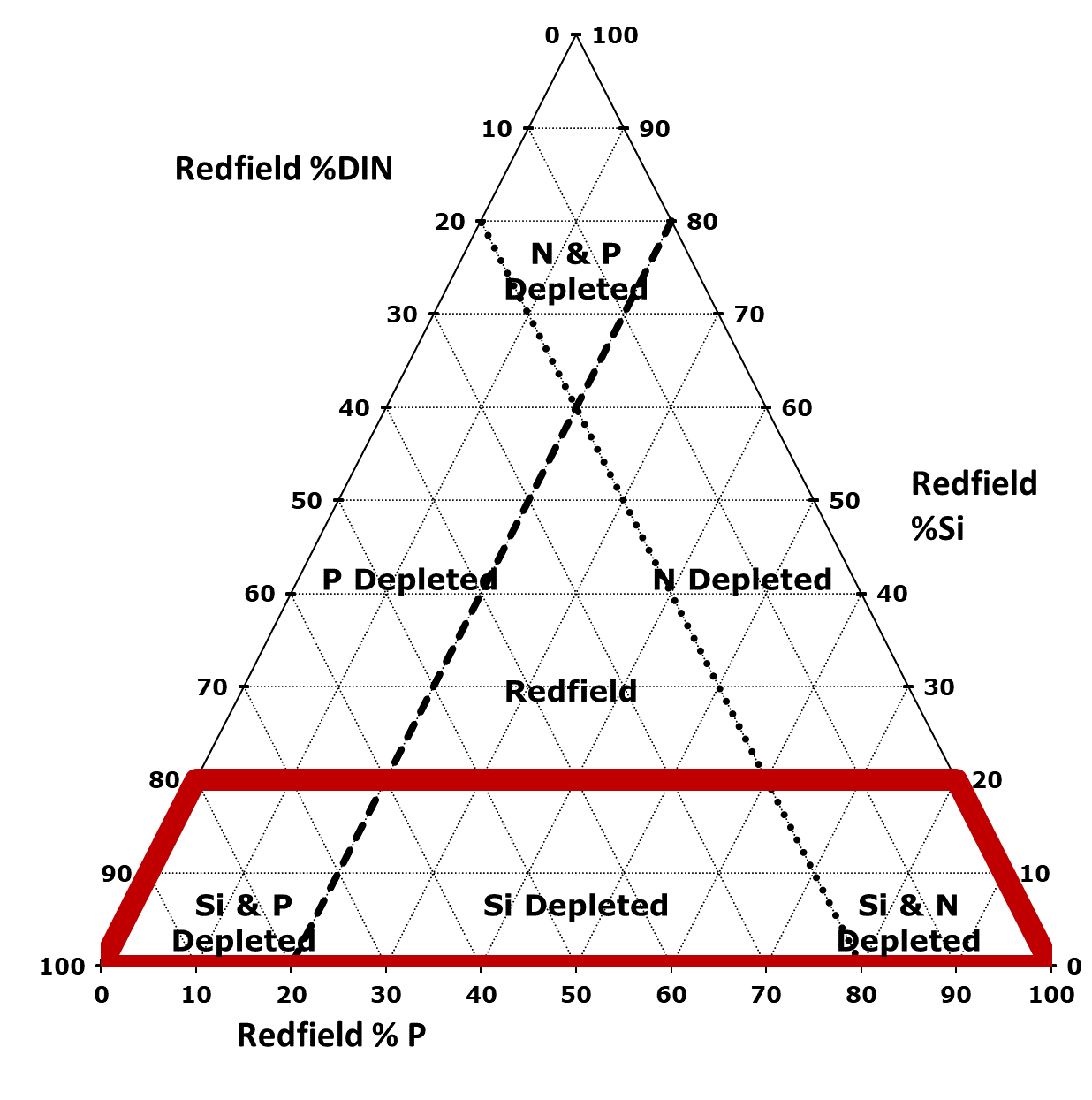 | 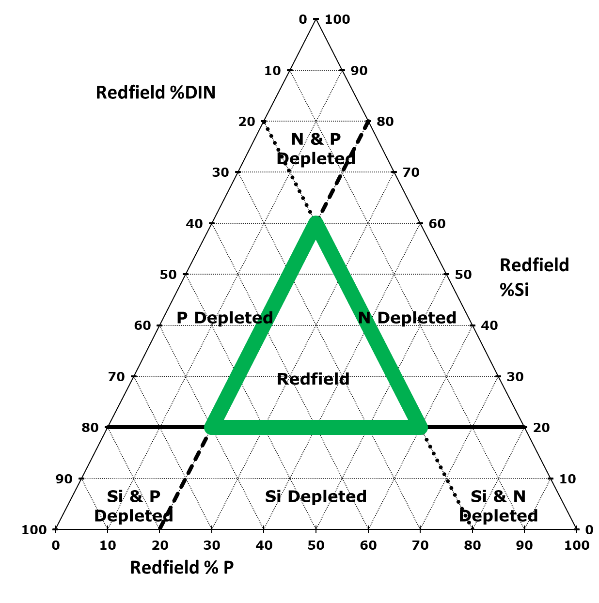 |

Supplementary Figure 1: Ternary diagram for representing N:P:Si Redfield ratios showing zones of relative nutrient depletion. The point at the centre of the ternary diagram (33.33, 33.33, 33.33) corresponds to the Redfield molar ratio of N:P:Si = 16:1:20. The horizontal lines represent the proportion of Si in a sample scaled to the Redfield ratio (Redfield %Si). The diagonal lines running from bottom left to top right represent the proportion of total phosphorus in a sample relative to the Redfield ratio (Redfield %P). The diagonal lines running from bottom right to top left represent the proportion of inorganic nitrogen in a sample scaled to the Redfield ratio (Redfield %DIN). The upper left panel (orange lines) shows the zone of relative P depletion. The upper right panel (blue lines) shows the zone of relative N depletion while the lower left panel (red lines) shows the zone of relative Si depletion. The lower right panel (green lines) shows the zone of “balanced” nutrient concentrations.

Supplementary Figure 2: Nutrient stoichiometry from Nordic rivers (grey dots), UK Rivers (purple) have been extracted from Table 3 of Tye et al. 2024), Latvian (LA), Danish (DK), Lithuanian (LV) and Russian (RU) rivers (green). The Nemunas (LA) and Neva (RU) are major riverine inputs to the Eastern Baltic.

Supplementary Table 1: Locations, names, average dissolved inorganic nitrogen (DIN), total phosphorus (TotP) and silicon (Si) concentrations and trophic status indices (TSI) for river mouths included in this study. All concentrations are expressed in units of µg/l. Latitude and longitude are expressed as decimal degrees.

| **ID** | **Location** | **Country** | **Latitude** | **Longitude** | **DIN** | **TotP** | **Si** | **TSI** |
| --- | --- | --- | --- | --- | --- | --- | --- | --- |
| 1 | Ähtävänjoki | FI | 63.640 | 22.816 | 423.5 | 49.7 | 2442.5 | 43 |
| 2 | Aurajoki | FI | 60.476 | 22.362 | 1516.5 | 146.9 | 5201.7 | 67 |
| 3 | Eurajoki | FI | 61.201 | 21.726 | 1211.5 | 39.0 | 4486.8 | 56 |
| 4 | Kalajoki | FI | 64.258 | 23.942 | 839.5 | 64.8 | 5268.3 | 61 |
| 5 | Kiiminkijoki | FI | 65.180 | 25.356 | 96.0 | 32.7 | 4102.7 | 17 |
| 6 | Kisko 14 Vanhakajoki | FI | 60.133 | 23.154 | 368.7 | 57.4 | 2404.5 | 39 |
| 7 | Koskenkylänjoki | FI | 60.500 | 25.942 | 1000.8 | 100.7 | 3706.0 | 64 |
| 8 | Lapuanjoki | FI | 63.525 | 22.528 | 1069.2 | 63.0 | 5924.4 | 63 |
| 9 | Lestijoki | FI | 64.063 | 23.655 | 391.6 | 51.9 | 3923.2 | 43 |
| 10 | Mustijoki | FI | 60.369 | 25.563 | 1372.1 | 92.4 | 5216.5 | 62 |
| 11 | Mustionjoki | FI | 60.087 | 23.638 | 291.0 | 30.4 | 1396.5 | 47 |
| 12 | Myllykanava | FI | 62.253 | 21.510 | 399.8 | 56.0 | 5735.7 | 43 |
| 13 | Pajo 44 Isosilta | FI | 60.462 | 22.677 | 1586.0 | 175.4 | 4482.6 | 62 |
| 14 | Perhonjoki | FI | 63.854 | 23.220 | 545.9 | 59.4 | 4343.1 | 60 |
| 15 | Porvoonjoki | FI | 60.442 | 25.608 | 2087.8 | 99.9 | 4866.8 | 69 |
| 16 | Pyhäjoki | FI | 64.462 | 24.267 | 435.8 | 49.2 | 4210.6 | 54 |
| 17 | Siikajoki | FI | 64.718 | 24.960 | 431.2 | 65.0 | 5078.3 | 41 |
| 18 | Skatila | FI | 63.093 | 21.882 | 1520.6 | 69.8 | 6451.3 | 65 |
| 19 | Vantaa | FI | 60.237 | 24.985 | 1245.9 | 82.0 | 4834.3 | 66 |
| 20 | Virojoki | FI | 60.584 | 27.709 | 341.4 | 47.7 | 3980.4 | 38 |
| 21 | Drammenselva | NO | 59.754 | 10.009 | 266.4 | 7.2 | 1395.4 | 30 |
| 22 | Skienselva | NO | 59.199 | 9.611 | 141.6 | 4.4 | 1044.8 | 24 |
| 23 | Otra | NO | 58.187 | 7.954 | 77.7 | 3.8 | 724.4 | 22 |
| 24 | Numedalslågen | NO | 59.086 | 10.070 | 250.8 | 12.8 | 1675.7 | 37 |
| 25 | Glomma | NO | 59.280 | 11.134 | 366.8 | 16.6 | 1719.2 | 42 |
| 26 | Orkla | NO | 63.201 | 9.773 | 196.8 | 4.5 | 1440.9 | 24 |
| 27 | Altaelva | NO | 69.901 | 23.287 | 50.5 | 6.6 | 2423.3 | 25 |
| 28 | Vefsna | NO | 65.749 | 13.239 | 53.4 | 3.0 | 732.2 | 13 |
| 29 | Orreelva | NO | 58.731 | 5.529 | 637.5 | 68 | 1013.2 | 33 |
| 30 | Pasvikelva | NO | 69.501 | 30.116 | 47.3 | 5.7 | 2437.3 | 4 |
| 31 | Tanaelva | NO | 70.230 | 28.174 | 51.8 | 5.5 | 3690.8 | 4 |
| 32 | Vosso | NO | 60.647 | 6.112 | 108.9 | 3.3 | 478.7 | 20 |
| 33 | Driva | NO | 62.669 | 8.571 | 165.1 | 3.8 | 1462.4 | 19 |
| 34 | Bjerkreimselva | NO | 58.479 | 5.995 | 337.1 | 4.9 | 711.3 | 25 |
| 35 | Vikedalselva | NO | 59.500 | 5.910 | 217.1 | 4.5 | 459.4 | 23 |
| 36 | Nausta | NO | 61.517 | 5.723 | 80.5 | 5.3 | 551.6 | 25 |
| 37 | Nidelva (Trondheim) | NO | 63.433 | 10.407 | 95.0 | 4.9 | 991.2 | 24 |
| 38 | Målselva 1 | NO | 69.036 | 18.666 | 63.9 | 4.5 | 1271.4 | 18 |
| 39 | Vegårdselva | NO | 58.670 | 8.981 | 144.4 | 7.6 | 1195.5 | 33 |
| 40 | Alna | NO | 59.905 | 10.791 | 1064.5 | 75.3 | 3114.7 | 65 |
| 41 | Målselva 2 | NO | 69.139 | 18.604 | 84.2 | 6.8 | 1162.8 | 26 |
| 43 | Alsterån | SE | 57.009 | 16.162 | 116.8 | 13.6 | 2201.8 | 16 |
| 44 | Alterälven | SE | 65.420 | 21.486 | 79.1 | 26.0 | 4141.5 | 13 |
| 45 | Ångermanälven | SE | 63.172 | 17.264 | 50.0 | 6.2 | 1596.8 | 19 |
| 46 | Ätran | SE | 56.903 | 12.495 | 623.0 | 18.2 | 2694.8 | 45 |
| 47 | Bäveån | SE | 58.347 | 11.939 | 335.0 | 42.7 | 2200.8 | 50 |
| 48 | Botorpströmmen | SE | 57.664 | 16.496 | 154.7 | 14.0 | 1751.5 | 27 |
| 51 | Dalälven | SE | 60.564 | 17.441 | 124.9 | 11.9 | 2354.3 | 20 |
| 52 | Delångersån | SE | 61.638 | 17.086 | 101.0 | 7.9 | 2410.4 | 30 |
| 53 | Emån | SE | 57.141 | 16.453 | 359.5 | 14.5 | 2788.2 | 42 |
| 54 | Enningdalsälv | SE | 58.877 | 11.537 | 213.0 | 10.6 | 1622.7 | 38 |
| 55 | Forsmarksån | SE | 60.351 | 18.205 | 128.4 | 19.3 | 2821.7 | 22 |
| 56 | Gavleån | SE | 60.674 | 17.127 | 228.8 | 27.2 | 1906.1 | 37 |
| 57 | Gideälven | SE | 63.346 | 19.116 | 51.0 | 11.7 | 3149.5 | 9 |
| 58 | Göta älv | SE | 57.764 | 12.002 | 384.3 | 17.3 | 723.2 | 44 |
| 59 | Göta älv | SE | 58.284 | 12.281 | 369.0 | 10.2 | 520.4 | 37 |
| 60 | Gothemsån | SE | 57.558 | 18.599 | 3445.4 | 40.6 | 1950.8 | 56 |
| 61 | Helge å | SE | 55.943 | 14.219 | 729.0 | 38.6 | 3140.0 | 56 |
| 62 | Indalsälven | SE | 62.525 | 17.399 | 84.6 | 4.5 | 1070.3 | 23 |
| 63 | Kalix älv | SE | 65.840 | 23.173 | 130.3 | 14.0 | 3286.2 | 29 |
| 64 | Kävlingeån | SE | 55.780 | 13.077 | 3220.7 | 66.9 | 3541.7 | 64 |
| 65 | Lagån | SE | 56.516 | 13.052 | 304.9 | 19.5 | 2396.5 | 47 |
| 66 | Ljungån | SE | 62.364 | 16.961 | 55.6 | 5.4 | 2419.3 | 25 |
| 67 | Ljungbyån | SE | 56.632 | 16.173 | 1260.9 | 20.2 | 4471.9 | 47 |
| 68 | Ljusnån | SE | 61.213 | 17.082 | 78.6 | 7.7 | 3061.5 | 31 |
| 69 | Lögdeälven | SE | 63.552 | 19.415 | 43.7 | 21.7 | 3763.5 | 6 |
| 70 | Lule älv | SE | 65.601 | 22.010 | 33.0 | 6.5 | 1497.6 | 2 |
| 71 | Lyckebyån | SE | 56.199 | 15.663 | 233.6 | 27.2 | 3585.6 | 45 |
| 72 | Mörrumsån | SE | 56.189 | 14.750 | 224.9 | 22.5 | 1203.3 | 43 |
| 73 | Motala ström | SE | 58.593 | 16.121 | 353.7 | 33.5 | 1298.6 | 44 |
| 74 | Nissan | SE | 56.691 | 12.874 | 433.7 | 20.7 | 3399.5 | 47 |
| 75 | Nordre älv | SE | 57.843 | 11.920 | 384.9 | 16.3 | 701.3 | 43 |
| 79 | Nyköpingsån | SE | 58.831 | 16.921 | 153.8 | 38.4 | 746.6 | 15 |
| 80 | Öreälven | SE | 63.702 | 19.596 | 66.0 | 18.2 | 3917.1 | 12 |
| 81 | Örekilsälven | SE | 58.460 | 11.686 | 415.3 | 35.7 | 2353.4 | 55 |
| 82 | Pite älv | SE | 65.379 | 21.299 | 54.4 | 10.7 | 2774.7 | 6 |
| 83 | Råån | SE | 56.000 | 12.779 | 6041.6 | 106.7 | 4468.0 | 70 |
| 84 | Råneälven | SE | 66.021 | 21.968 | 37.7 | 11.4 | 4010.4 | 4 |
| 85 | Rickleån 1 | SE | 64.192 | 20.849 | 72.1 | 11.0 | 3077.8 | 18 |
| 86 | Rickleån 2 | SE | 64.087 | 20.941 | 117.6 | 14.8 | 3241.9 | 28 |
| 87 | Rönneån | SE | 56.122 | 13.145 | 1242.5 | 36.2 | 3719.8 | 55 |
| 89 | Skellefteälven | SE | 64.740 | 20.768 | 43.3 | 4.4 | 1575.0 | 24 |
| 90 | Skivarpsån | SE | 55.449 | 13.593 | 4275.7 | 117.0 | 4898.8 | 72 |
| 91 | Smedjeån | SE | 56.510 | 12.975 | 3243 | 61.4 | 4810.9 | 61 |
| 92 | Stockholm Centralbron | SE | 59.327 | 18.061 | 106.1 | 24.8 | 529.5 | 15 |
| 93 | Töreälven | SE | 65.938 | 22.640 | 29.8 | 22.8 | 3918 | 0 |
| 94 | Torne älv | SE | 65.875 | 24.131 | 67.7 | 15.5 | 3649.8 | 6 |
| 95 | Ume älv | SE | 63.853 | 20.049 | 46.7 | 6.7 | 1645.3 | 5 |
| 96 | Viskån | SE | 57.239 | 12.309 | 620.6 | 28.2 | 2521.9 | 51 |
